# Strategic soft power for developing poultry production competencies and empowering the next generation of industry professionals

**DOI:** 10.1016/j.psj.2025.105805

**Published:** 2025-09-07

**Authors:** Farid S. Nassar

**Affiliations:** Department of Animal and Fish Production, College of Agricultural and Food Sciences, King Faisal University, Al-Ahsa, Saudi Arabia

**Keywords:** Food Security, Interpersonal skills, Poultry production, Production competencies, Sustainability

## Abstract

The poultry industry plays a crucial role in addressing global challenges, which necessitates the modernization of educational programs to develop not only technical competencies but also global and social awareness for sustainable advancement. Extracurricular activities (ECA) have gained wide recognition as essential tools for fostering holistic personal and professional development, particularly within academic programs. While formal education provides the foundation for technical knowledge, ECA offers students valuable opportunities to enhance their professional and interpersonal skills. These activities serve as a vital complement to in-class learning. This study aims to explore the role of ECA as a form of strategic soft power in developing the professional and personal competencies of students enrolled in poultry science programs and in preparing them to meet the future challenges of the industry. A qualitative descriptive methodology was adopted, including a review of relevant literature, to assess the impact of employing ECA as a strategic soft power in shaping the competencies of poultry science students within higher education institutions (HEIs). The study indicates that the effective use of extracurricular activities (ECA) in poultry science programs is expected to have a positive impact on student retention rates and enhance the programs’ attractiveness, thereby encouraging more students to enroll. Additionally, these activities play a central role in offering graduates valuable opportunities to strengthen their professional and personal skills, enabling them to meet the dynamic and growing demands of the poultry industry. Based on these insights, the study recommends integrating ECA into poultry science curricula as a core component of student development. The HEIs should allocate adequate resources to support such initiatives and foster partnerships with industry stakeholders to offer training opportunities and practical workshops. Moreover, HEIs should cultivate an inclusive environment that encourages active student participation in extracurricular engagements, thereby enhancing their employability and leadership potential within the poultry sector.

## Introduction

Poultry production plays a critical role in addressing major global challenges such as biodiversity loss, climate change, and food insecurity, underscoring the strategic importance of this sector in advancing global sustainability. Consequently, the education of undergraduate poultry science students must extend beyond traditional technical training to cultivate professionals who possess global awareness and social responsibility. This highlights the urgent need to develop poultry science programs that align with current global priorities (Fanatico et al., 2025). Consequently, careers within the poultry sector are constantly adapting to address emerging global challenges, driving employers to prioritize hiring university graduates who can implement innovative solutions to both local and global industry demands. Thus, undergraduate programs in animal and poultry sciences must prioritize curricula and experiential opportunities that equip students with the skills and experiences necessary for workforce readiness and professional growth. Notably, the 2024 Poultry Science Association annual conference included a symposium that presented insightful viewpoints from leading academics and industry professionals regarding the essential competencies graduates need to thrive in this field (Karcher et al., 2025).

Moreover, graduates of poultry science programs significantly contribute to community development by supporting localized food systems that retain economic value within regional economies and strengthen the connection between consumers and their food sources. When consumers know their farmers, they become more informed and invested in sustainable agricultural practices. By integrating academic knowledge with hands-on experience, these graduates not only meet immediate industry needs but also actively participate in broader global dialogues on sustainability in poultry production. Consequently, they emerge as informed, engaged citizens capable of driving meaningful change in an increasingly interconnected world (Fanatico et al., 2025).

Employers increasingly face challenges in recruiting qualified agricultural professionals, largely due to noticeable skill gaps among graduates entering the workforce. While subject-matter expertise and technological proficiency were once the primary criteria for evaluating candidates in the agricultural sector, hiring decisions today are more heavily influenced by the presence of employability and behavioral skills rather than academic achievements alone(Parrella et al., 2024). As a result, graduates from agricultural colleges are now expected to combine strong technical competencies with advanced soft and transferable skills in order to meet the evolving demands of the industry (Parrella et al., 2024).

Furthermore, there is a growing and unmistakable demand from employers for universities to implement more effective and proactive strategies in preparing graduates to meet workforce challenges and adapt to rapidly evolving requirements. Employers emphasize that many of the desired qualities in graduates cannot be cultivated solely through traditional academic instruction but require active engagement in extracurricular activities (ECA). Such efforts are crucial not only for directly enhancing the academic reputation of institutions but also for attracting a larger number of high-achieving students. This progress manifests through improved academic performance, richer university experiences, a stronger sense of belonging within the academic community, development of intellectual capital, and acquisition of a diverse skill set that significantly boosts graduates’ employability (Nassar et al., 2024).

Accordingly, Higher education institutions (HEIs) are therefore tasked with preparing graduates who possess the skills and competencies required to successfully adapt to the rapid changes brought about by globalization and digital transformation (Cawood et al., 2018). Graduate employability has increasingly become a pivotal concern shaping educational policies and institutional practices (Smith et al., 2018). This has led to the ongoing growth of initiatives centered on employability (Bennett, 2018) and a strong, continuous focus on improving students’ readiness for the workforce. The design and implementation of these initiatives are typically guided by each institution’s strategic priorities related to employability, which may include achieving immediate employment outcomes, fostering professional preparedness, and encouraging lifelong learning (Bridgstock and Jackson, 2019).

The prevailing perspective on graduate employability underscores the vital role of ECA within academic programs at HEIs in developing essential skills, competencies, and attributes that significantly contribute to individuals’ ability to secure and maintain meaningful employment post-graduation (Tran, 2017). Additionally, Isopahkala-Bouret et al. (2023) found that social networks formed within HEIs through participation in student clubs play a positive role in enhancing graduate employability.

On the other hand, HEIs through their extracurricular programs, create safe environments where students can explore social innovation and entrepreneurship without the fear of real-world failure (Bodolica et al., 2021). While some HEIs offer occasional extracurricular opportunities, these initiatives often lack depth due to insufficient sustained commitment and seriousness. Many such activities remain predominantly instructor-led, limiting students to passive roles and excluding them from the design and execution of the programs. Providing robust support for foundation program students via ECA is crucial, as these programs establish a unique framework for success characterized by structured methodologies, active student participation, and a strong focus on holistic and independent learning (Ginosyan et al., 2020). Undeniably, ECA offers extensive benefits, with students frequently recognizing them as vital support across multiple dimensions of their academic and personal development (Ginosyan et al., 2020).

This study explores the role of ECA as a strategic soft power in developing poultry production competencies and empowering the next generation of industry professionals to meet the demands of the labor market and stakeholders. The study addresses three main questions:

Q1- Do ECA serve as a bridge between academic education and labor market requirements in the poultry industry?
Q2- Does the soft power of extracurricular activities contribute to preparing poultry industry professionals and enhancing the quality of poultry science programs?
Q3- What are the challenges and obstacles facing the implementation of ECA in developing students' skills in the field of poultry production, and how can they be overcome?

In light of the intensifying global competition among poultry science programs in higher education institutions, and the ongoing efforts to adopt diverse strategies aimed at enhancing student experience and strengthening the academic reputation of these programs—particularly in achieving their objectives of developing poultry production competencies and empowering the next generation of industry professionals it has become imperative to conduct a comprehensive evaluation of the implementation of extracurricular activities (ECA) as a strategic form of soft power to achieve these vital goals. This study aims to provide an in-depth analysis of the role of ECA as a strategic tool in significantly enhancing the student experience within poultry science programs, while simultaneously elevating academic standards and program quality. Such efforts directly contribute to the development of professional competencies and the empowerment of future leaders in the poultry industry. Furthermore, this study seeks to address a critical knowledge gap in academic literature by offering a timely and relevant resource on a topic of growing global importance.

However, despite the increasing recognition of the vital role of extracurricular activities in developing students’ essential skills and competencies, there remains a clear gap in the research literature regarding the evaluation of ECA as a strategic soft power tool within poultry science programs. This gap is particularly evident in understanding how extracurricular activities can be effectively integrated into the academic and strategic frameworks of undergraduate programs, and in assessing their impact on improving student experience, enhancing production competencies, and empowering the next generation of industry professionals.

In other words, while previous studies have highlighted the general benefits of extracurricular activities in improving employability and developing personal skills, research that directly examines the application of ECA as a strategic tool specifically within poultry science programs is extremely limited. This shortage underscores the urgent need for a comprehensive study that evaluates the actual impact of extracurricular activities at the student, program, and labor market levels that this study aims to address by providing a clear analytical framework for understanding the role of ECA as a strategic soft power tool in developing competencies and empowering future leaders in the poultry industry.

## Material and methods

This study employs a qualitative descriptive research methodology to examine the role of extracurricular activities (ECA) as a form of strategic soft power in fostering poultry production competencies and empowering the next generation of professionals in the poultry industry. Furthermore, descriptive research, as described by Neuman (2014), is characterized by its ability to “present a picture of the specific details of a situation, social setting, or relationship” and “begins with a well-defined issue or question and endeavors to describe it accurately.” & “…concentrates on ‘how’ and ‘who’ inquiries…” . Lambert and Lambert (2012) contend that qualitative research designs, including phenomenology and grounded theory, can serve both descriptive and explanatory purposes. They suggest that the term "qualitative descriptive research" may be employed to accurately characterize the research approach, rather than mislabeling it with terms associated with other methodologies (e.g., phenomenology, grounded theory, ethnography). Lambert and Lambert (2012). Qualitative descriptive research "…often derives from naturalistic inquiry, which asserts a dedication to examining phenomena in their inherent state as much as feasible within the research context" (Lambert and Lambert, 2012). The study, inherently qualitative, seeks to examine the role of ECA as a form of strategic soft power in fostering poultry production competencies and empowering the next generation of professionals in the poultry industry; hence, it constitutes qualitative descriptive research. Furthermore, given the objectives of this study, a comprehensive content analysis of both electronic and printed materials related to the events under investigation is essential (Bowen, 2009). According to Bowen (2009), content analysis involves three key phases: skimming, thorough reading, and interpretation. This method enables the systematic categorization of textual data into smaller, meaningful, and manageable units (Weber, 1990), with the aim of uncovering underlying meanings by identifying recurring themes and patterns within the sources (Patton, 2002).

The methodology adopted in this study followed a structured, four-phase approach. In the first phase, a comprehensive systematic search was conducted across leading academic databases, including Google Scholar, Scopus, and Web of Science, using a targeted set of keywords such as “Extracurricular Activities,” “Poultry Production,” “Higher Education,” “Soft Skills Development,” and “Labor Market''. The search focused on peer-reviewed articles, institutional reports, and empirical studies published in English over the past five years, supplemented by foundational literature to provide comprehensive scholarly support.

The second phase involved defining precise inclusion and exclusion criteria. Studies were included if they directly addressed the role of ECA in enhancing educational and professional outcomes for higher education students, particularly in relation to the development of practical, job-ready skills relevant to production-oriented environments such as factories and livestock farms. Studies were excluded if they lacked a clear connection to higher education or production settings, or if they were methodologically weak or lacked empirical evidence. During the third phase, data were extracted and analyzed from the selected sources, focusing on the main objectives of each study, key findings, the role of ECA in higher education, identified challenges, and proposed recommendations. A qualitative synthesis of the data were conducted to uncover the core benefits of extracurricular engagement and to determine its strategic contribution to improving the quality of poultry science programs and developing students’ professional competencies within the poultry production sector. The fourth and final phase comprised an in-depth analysis of the findings to explore how ECA can serve as a form of strategic soft power within an integrated educational framework. This phase aimed to demonstrate how these activities enhance student learning experiences, improve academic program quality, and strengthen graduate competencies both within HEIs and across the poultry industry. The findings were thoroughly discussed, and future opportunities were explored for advancing academic program effectiveness, developing poultry production capacities, and empowering the next generation of professionals in alignment with the evolving demands of the labor market. This structured methodological framework was designed to provide a rigorous and comprehensive assessment of the role of ECA in supporting HEIs and preparing a new generation of highly qualified professionals in the poultry sector through the lens of strategic soft power.

## Results and discussion

### ECA as an effective bridge between academic education and labor market requirements in the poultry industry

A recent report by the National Institute of Food and Agriculture under the U.S. Department of Agriculture indicates that graduates of degree programs in Food, Agriculture, Renewable Natural Resources, and the Environment are expected to comprise approximately 61 % of the new entrants to the job market in these sectors, while the remaining 39 % will come from related or unrelated fields (Fernandez et al., 2020). Moreover, the skills gaps between university graduates and employer needs in the agricultural sector are widening, particularly among recent graduates, potentially leading to negative consequences in the poultry industry. These consequences include weak engagement, high employee turnover, and occupational burnout. Furthermore, such issues may impose increased costs on employers due to decreased productivity and higher training expenses for employees who do not remain in the job long term. Understanding the implications of these skills gaps and mitigating their impact is critical when hiring students for various roles within the poultry industry (Karcher et al., 2025).

On the other hand, Bundy et al., 2021, indicated that interpersonal and teamwork skills are most critical for success in entry-level roles within the animal industry, alongside the importance of animal science coursework and hands-on experience with animal care and welfare. While non-animal science and some scientific and management-related skills were rated as moderately important, they remain essential. This underscores the need for HEIs to produce well-rounded graduates who combine technical and soft skills, emphasizing the integration of projects, written assignments, and experiential activities that foster soft skill development within animal science curricula.

The prevailing understanding of graduate employability underscores the essential role of ECA within higher education curricula. Such activities facilitate the development of critical skills, competencies, and personal attributes that equip graduates to successfully enter and sustain employment in their respective fields (Tran, 2017). Moreover, findings from Isopahkala-Bouret et al. (2023) indicate that social networks cultivated through participation in these activities within university settings significantly enhance graduates’ employment prospects.

Researchers have defined ECA in various ways. Belikova (2002) described them as activities occurring "during time free from schoolwork," while Christensen et al. (2002) referred to them as "outside-classroom activities." Nelson et al. (2002) called them "beyond-classroom activities"; Chia (2005) labeled them "non-academic pursuits," and Mahoney et al. (2003) described them as “organized, challenging, and voluntary". Moreover, Cole et al. (2007) emphasized ECA as a vital part of the educational experience, offering students the opportunity to develop leadership and interpersonal communication skills. This study adopts the definition by Bartkus et al. (2012), which states: “ECA are academic or non-academic activities conducted under the school’s supervision but outside formal class time and not part of the official curriculum. Furthermore, they do not offer grades or academic credit, and participation is voluntary.

On the other hand, ECA play a fundamental role in promoting active learning among students by motivating them to pursue knowledge beyond exams. ECAs meet three essential criteria for fostering active learning: they are typically voluntary, driven by intrinsic student motivation; they occur in a non-evaluative environment that encourages safe exploration; and they utilize non-traditional methods offering a wide array of student-tailored activities based on individual interests and learning styles. Their core goals align with the key outcomes of active learning, particularly learner autonomy and skills development (Ginosyan et al., 2020).

Through extracurricular involvement, students gain access to supportive mentoring relationships and receive constructive feedback that enhances their learning curve (Aliu and Aigbavboa, 2020). These activities also allow students to explore talents and capabilities beyond the classroom, cultivate a sense of commitment, foster friendships, boost self-confidence, and may enrich résumés. Additionally, they provide opportunities for acquiring new knowledge through interaction with peers and professionals from diverse academic, social, and cultural backgrounds—broadening perspectives through recreational engagement (Aliu and Aigbavboa, 2023).

Thompson et al. (2013) highlight a broad spectrum of ECA examples such as hobbies, social groups, sports, cultural or religious activities, and volunteer or paid work, emphasizing that to qualify as extracurricular, activities must possess both “communal interest/value” and “structure/organization.” Others suggest that ECAs are often facilitated within HEIs and have called for a broader definition that includes both on-campus and off-campus activities falling outside the formal curriculum (King et al., 2021). The number and variety of ECA opportunities provided by institutions reflect stakeholders’ recognition of their value (Stuart et al., 2009). Evidence supports students' awareness of ECA importance, including their strategic participation to distinguish themselves in competitive job markets (Roulin and Bangerter, 2013).

Moreover, ECA helps students acquire job relevant skills and knowledge. Student clubs, for instance, are a powerful means of shaping independent, creative, and in-demand professionals within modern education systems. These clubs not only deepen specialized knowledge but also play a significant role in personal growth. International studies suggest that educational programs rooted in creativity and practical engagement enhance mastery and prepare students for the demands of a market-driven economy. Student clubs offer a unique learning environment that fosters individual engagement, covering not only professional skills but also social competencies such as adaptability, teamwork, aesthetic appreciation, and responsibility. Moreover, students’ structured participation in organized and purpose-driven ECA enhances their motivation, fosters a sense of responsibility, and significantly enriches their professional competencies (Tojimukhammadovna, 2025).

Higher education students are increasingly expected to demonstrate value beyond academic achievement to succeed in a competitive job market. Four main motivational factors drive student participation in ECAs: extrinsic, intrinsic, social, and prosocial motivations (Chapman et al., 2023). These differ by activity type, extrinsic motivations dominate employment-related, academic, association, and volunteer activities, while intrinsic and social motivations are more evident in sports and association contexts. Prosocial motivations are particularly important in academic, volunteer, and association activities. Moreover, notable differences have been observed between early-year and final-year students’ motivations, indicating a developmental trajectory in ECA participation that aligns with workforce readiness (Chapman et al., 2023).

In light of the growing emphasis on enhancing employability skills among students in the agricultural sector, Crawford and Fink (2020) reported that feedback from students, faculty members, alumni, and employers across agricultural institutions showed that fundamental skills such as active listening, clear and concise communication, and problem analysis are top priorities. The most significant gaps in student readiness were found in advanced competencies, including understanding job expectations, constructive conflict resolution, and the ability to accept feedback. Moreover, both employers and faculty identified specific co-curricular activities essential for developing employability skills, such as part-time jobs, field-based internships, research participation, and volunteer work (Karcher et al., 2025).

In this context, Parrella et al. (2024) conducted a study at Texas A&M University’s College of Agriculture and Life Sciences to assess students’ awareness of employability skills, their development, and the factors influencing this growth. They Reported that students regard communication and decision-making as the most essential employability skills, while teamwork and self-management were identified as the most developed throughout their academic journey. Furthermore, students with clearly defined career goals demonstrated significantly greater skill development compared to their peers lacking a clear professional direction. Continuous participation in activities such as teamwork, leadership, project management, community service, interdisciplinary initiatives, international programs, and practical training was shown to have a positive impact on enhancing students’ human capital. Clearly articulated career pathways were recognized as a crucial factor in effectively building and sustaining this human capital over time.

On the other hand, Meyer and Bobeck (2023) indicated that personal backgrounds and career aspirations play a significant role in shaping students’ perspectives on current issues in the poultry industry. This underscores the importance of developing flexible curricula that are responsive to such diversity. It also highlights the need for educators and industry professionals to thoughtfully integrate both individual experiences and scientific principles when designing educational initiatives aimed at students and consumers, in order to effectively support and advance the poultry sector.

York (2006) defines employability as “a set of achievements –skills, understandings, and personal attributes –that make graduates more likely to gain employment and succeed in their chosen occupations, benefiting themselves, the workforce, the community, and the economy.” There is a positive relationship between participation in ECA and the development of transferable skills that enhance employability, such as communication, teamwork, and project management (Wood et al., 2011). Moreover, students’ involvement in ECA can have a broader impact by playing key roles in “whole-person education” (Chan, 2016) and “life-wide learning” (Jackson, 2008). Participation in such activities provides contextual benefits that help students better understand the broader applications of their academic studies by exploring the link between involvement in these activities and increased levels of self-efficacy specific to the university context (Griffiths et al., 2021).

On the other hand, the ECA plays a pivotal role in complementing traditional academic education by fostering personal and professional skills that are often difficult to develop through theoretical lectures alone. In the poultry production sector, which demands advanced technical and practical competencies, these activities serve a crucial function in bridging the gap between higher education outcomes and the evolving requirements of the labor market. This study highlights the importance of ECA in preparing poultry science students and enhancing their professional readiness by enabling them to acquire skills such as leadership, teamwork, and problem-solving, alongside real-world exposure to industry challenges. It also addresses the existing skills gaps between graduates and employers, presenting evidence that active participation in ECA significantly improves employability. The study concludes by emphasizing the need for HEIs to adopt integrative educational strategies that enhance soft skills development and support the sustainable professional growth of students, ultimately contributing to a qualified workforce capable of meeting the demands of the modern poultry industry.

### The soft power of ECA in preparing poultry industry professionals and enhancing the quality of poultry science programs

Amid the rapid growth of the poultry industry today, there is an increasing demand for professionals equipped with advanced technical knowledge and essential personal skills that enable them to succeed in this vital sector. While traditional academic curricula provide students with a solid scientific and technical foundation, they often fall short in developing the soft skills and practical competencies necessary to meet the actual demands of the labor market. Extracurricular activities, regarded as a form of “soft power,” play a pivotal role in bridging this gap by enhancing leadership, teamwork, communication, and problem-solving skills, thereby fostering the personal and professional growth of students in a comprehensive manner. This skills development not only prepares graduates effectively but also contributes to elevating the quality and academic reputation of poultry science programs. Student participation in ECA is considered a strategic tool that prepares flexible and qualified professionals capable of facing the dynamic challenges of the poultry industry. It strengthens the alignment between educational outcomes and labor market needs and enhances the academic standing of these programs by cultivating practical skills, adaptive abilities, and social competencies required in the workforce.

***The Soft Power of ECA in Academic Performance.*** Over the past few decades, growing recognition that student success cannot be documented as is usually done solely in terms of enrollment, persistence, and degree attainment has fueled increasing academic interest in the role of ECAs in enhancing both academic outcomes and the personal and professional development of university students. A substantial body of research has demonstrated that ECAs offer significant benefits in terms of student enrollment and persistence through graduation (Kahu and Nelson, 2018). Moreover, positive correlations have been found between ECA participation and improvements in active and critical thinking, civic values, and cognitive development (Pascarella et al., 2014). These benefits extend to academic performance indicators such as higher GPA and increased knowledge acquisition (Zhu and Arnold, 2013). For instance, Webber et al. (2013) found that “higher levels of engagement in a variety of curricular and co-curricular activities significantly contribute to cumulative GPA and students’ perception of the overall academic experience”.Beyond academic performance, student success research highlights the vital role ECAs play in cultivating practical skills that are often underemphasized in the classroom. According to José Sá (2020) ECAs are not seen as a waste of time that could be used for academic activities, but rather as a complementary means of acquiring a set of competencies that students perceive as critical. Students often describe the balance between formal coursework and meaningful non-classroom learning as a defining feature of successful higher education experiences.

This perspective is especially relevant in animal and poultry science programs, where the presence and type of live animals have been shown to influence students’ academic performance, attitudes toward animals, knowledge retention, and physiological responses such as engagement and focus (George and Cole, 2018). At Ohio State University, for example, students exhibited increased concern for the welfare of agricultural and exotic animals featured in their coursework (George and Cole, 2018). On the other hand, in courses where direct interaction with live animals is not feasible, instructors are encouraged to guide students toward extracurricular opportunities such as career fairs or consultations with career services (Youngs et al., 2012). Virtual farm tours, when in-person experiences are unavailable, can also stimulate interest and provide meaningful real-world insights (Karcher and Reid, 2018).

In cases where formal research courses are lacking, structured extracurricular programs offer a viable alternative. Increased student participation in clubs and out-of-class activities has been linked to stronger engagement with animals, deeper connections with faculty and peers, and greater understanding of animal care and the scientific process. For example, students who participated in an Undergraduate Research Association in Animal Science reported improved application of course content, heightened animal engagement, and stronger peer relationships (Karcher and Trottier, 2014).

In parallel, Bandura (2012) defines academic self-efficacy as the belief in one’s learning effectiveness and the self-regulatory capacity to manage learning tasks that lead to academic achievement. This construct is vital, as it influences various aspects of student success in higher education from course engagement and distance learning participation (Tang and Tseng, 2013), to overall adjustment and satisfaction with academic experiences (Bowles et al., 2020). Moreover, improving self-efficacy enhances students’ ability to pursue short-, medium-, and long-term life goals (Lee and Vondracek, 2014). Participation in meaningful ECA with positive peer groups not only supports students’ wellbeing and quality of life but also helps them build essential personal capital, including self-efficacy and resilience (Griffiths et al., 2021). In the context of poultry science programs, student participation in ECA such as student clubs specializing in poultry production, hands-on workshops at educational farms, and poultry-related student competitions provides students with the opportunity to apply theoretical knowledge in practice. These activities enhance leadership and teamwork skills, develop self-efficacy in managing small-scale production projects, and contribute effectively to preparing students for the labor market while meeting the needs of the modern poultry industry.

On the other hand, Ribeiro et al. (2024) emphasized that HEIs should actively support and continuously promote ECA that extend beyond the formal curriculum and classroom settings. These initiatives are fundamental in fostering the holistic development of students by achieving an effective balance between academic excellence and professional readiness for the labor market. Furthermore, Griffiths et al. (2021) highlight the strong positive relationship between students’ engagement in ECA and their academic self-efficacy. This relationship not only impacts students' individual academic outcomes but also holds institutional significance, as it can translate into improved graduate employability rates an increasingly important performance indicator for HEIs themselves.

Moreover, ECA plays a pivotal role in enhancing students’ academic performance, as they have been shown to correlate with improved academic success indicators, strengthened critical thinking, and reinforced civic values, along with boosting self-confidence and self-regulation abilities. Integrating these activities into practical programs like animal and poultry sciences further increases student engagement and focus, positively impacting their academic achievement. Therefore, it is essential for HEIs and poultry science programs to invest in supporting and developing ECA to achieve an effective balance between academic excellence and students’ readiness for the labor market, ensuring the preparation of distinguished graduates capable of sustainable success and competitiveness.

***The Soft Power of ECA in Entrepreneurial Intention****.* The future of agriculture requires a workforce equipped with dynamic networks, innovative approaches, and proactive risk assessment capabilities (Alston et al., 2009). To meet these demands, twenty-first-century agricultural students must develop advanced behavioral skills that enable them to be effective collaborators, innovators, and problem-solvers addressing global challenges such as climate change, food security, and urbanization (Parrella et al., 2022). Juhász and Horváth-Csikós (2021) emphasized the critical role these skills play in student success across agricultural fields, underscoring the urgent need to integrate their development into postsecondary agricultural education programs. However, educators in higher agricultural education often focus on discipline-specific technical skills, neglecting to provide sufficient opportunities for students to develop essential behavioral competencies (Hendrix and Morrison, 2018).

Graduates of poultry science programs also contribute significantly to community development by supporting local food systems that maintain economic value within regional economies and strengthen the connection between consumers and their food sources. When consumers know their farmers, they become more aware of and invested in sustainable agricultural practices. By combining academic knowledge with practical experience, these graduates not only meet immediate industry needs but also actively participate in broader global discussions on sustainability in poultry production. Consequently, they emerge as informed and engaged citizens capable of driving meaningful change in an increasingly interconnected world (Fanatico et al., 2025).

Meanwhile, a growing body of research highlights the importance of entrepreneurship education in shaping students’ entrepreneurial intentions (Liñán and Fayolle, 2015). In response, universities have expanded their offerings to include a wide array of educational opportunities aimed at nurturing entrepreneurial mindsets, with ECA playing a pivotal role in this effort (Hien and Cho, 2018). These entrepreneurship-focused ECA consist of diverse actions, experiences, and innovations conducted both within and outside university settings, extending beyond the boundaries of formal academic curricula (Souitaris et al., 2007). They are designed to complement academic programs by sparking students’ interest in entrepreneurship, enhancing their entrepreneurial capabilities, and encouraging the creation of new ventures (Arranz et al., 2017). Examples of such activities include entrepreneurship simulation games, business plan competitions, international exchange programs, business mentoring, student clubs and societies, pre-incubation initiatives, skill-building workshops, entrepreneurship support services, innovation and product development contests, idea-generation events, and business incubators (Pittaway et al., 2011).

The ECA provides a supportive environment where students can actively engage in business and entrepreneurial experiences, thereby enhancing their awareness and attitudes toward entrepreneurship (Pittaway et al., 2011). These experiences strengthen students’ entrepreneurial intentions by building their knowledge and expanding their social networks (Peterman and Kennedy, 2003). Moreover, Nguyen et al. (2021) reported that participation in activities such as developing business plans, competing in contests, or joining entrepreneurship-focused student organizations positively impacts students’ motivation and intention to pursue entrepreneurial ventures.

The current study underscores the importance of integrating behavioral and entrepreneurial skills alongside technical competencies within poultry science programs. Developing these skills is essential to address escalating global challenges like food security and environmental sustainability. Activating the role of ECA, particularly those related to entrepreneurship, enhances students’ innovation and initiative, equipping them to deliver effective and creative solutions within the poultry sector. Consequently, poultry science programs that systematically incorporate these dimensions help prepare well-rounded graduates capable of making a positive impact on the industry and advancing it toward a sustainable future aligned with the rapidly evolving market and agricultural development demands.

***The Role of ECA in Enhancing Enrollment.*** Animal science departments have a legacy spanning over a century and remain integral to agricultural colleges due to their critical role in equipping students to meet the demands of a growing global population, work with diverse animal species, and engage in research with tangible applications (Lugar and Stewart, 2019). Graduates with degrees in animal science enjoy a broad spectrum of career opportunities (Benson et al., 2020). Nevertheless, these programs face the ongoing challenge of balancing theoretical scientific knowledge, research endeavors, and practical skill development. Furthermore, the student demographic within animal science has evolved, with a rising proportion of female students and a shift from primarily rural students focused on food animal production to more urban and suburban students who are increasingly oriented toward veterinary medicine (Peffer, 2011).

Student recruitment is crucial for the sustainability and success of academic programs, especially in applied fields like poultry science. Despite overall growth in university enrollment, poultry science programs have experienced stagnation or decline, raising concerns about the future of this discipline (Wells, 2019). Student numbers in agricultural colleges, particularly in poultry science, have not kept pace with the general expansion of higher education due to factors such as limited public awareness of the field’s importance, reduced institutional support, and geographic concentration of programs in key poultry-producing regions, which restricts expansion. Furthermore, the growing gap between the number of graduates and industry needs threatens the sustainability of the sector. Strategic actions are needed to enhance the appeal of discipline, update curricula, and align education with labor market demands (Wells, 2019).

Since the establishment of colleges, student clubs and organizations have played a pivotal role in shaping the university environment, with studies demonstrating their significant impact on enhancing academic performance and developing a wide range of student skills. In today’s increasingly competitive and market-driven higher education landscape, institutions attract students based on the quality of extracurricular experiences they offer, which address students’ psychological needs such as adaptation, stress relief, motivation, and engagement (Buckley and Lee, 2021). Moreover, marketing has become a crucial element in higher education, as institutions increasingly view students as “customers” and strive to provide education that leads to swift and effective employment opportunities (Elwick and Cannizzaro, 2017). However, ECA are now an integral part of campus life, involving students in community service and fostering a sense of significance and contribution to university development, which in turn enhances the institution’s academic standing. These benefits extend to both students and institutions, as universities aim to deliver high-quality education and a positive student experience that strengthens the sense of belonging. Graduates who feel proud of their alma mater are more likely to provide financial and moral support, promote the institution among peers and families, and serve as ambassadors in the wider community (Buckley and Lee, 2021).

The current emphasizes the necessity of adopting a comprehensive approach to developing poultry science programs that integrate scientific curricula, practical training, and ECA to meet the evolving needs of students and labor market demands. These programs face challenges such as declining student enrollment and reduced institutional support, requiring integrated strategies that raise awareness of the discipline’s importance and provide a stimulating educational environment that fosters both academic and social skill development. The study also highlights the crucial role of educational marketing in attracting students, fulfilling their professional aspirations, and preparing them for effective employment opportunities, enabling them to serve as ambassadors for the program within the community. This integration enhances the sustainability of the discipline, ensures the production of qualified graduates capable of meeting industry needs, and strengthens the relationship between graduates and educational institutions to support sustainable development in both academic and professional sectors.

***The Role of ECA in Preventing Early Student Dropout*.** The majority of academic attrition occurs during the early stages of undergraduate education, making it critically important to engage and motivate students from the outset. This challenge becomes even more complex in animal science programs, where the student demographic has shifted to include individuals from diverse backgrounds and experiences, many of whom have limited prior exposure to working with food-producing animals(Ragland et al., 2023). Moreover, the causes of student attrition are as diverse as the student population itself, including dropout, transfer, or temporary leave due to personal, financial, or socio-economic factors (Finnegan and Merrill, 2017). Additional causes involve mismatches between students and their chosen programs or institutions, as well as lack of academic or emotional preparedness (Jansen and van der Meer, 2012). One study noted that attrition was more likely among male students, those from rural areas, individuals with low engagement in high school homework, and students from certain Canadian provinces all of whom face higher risks of not completing their degrees (Shaienks et al., 2008).

The transition from high school to university poses a significant challenge; first year students are particularly vulnerable to dropping out, with nearly one fourth not returning for a second year. This life transition entails shifts in roles and social contexts, which can negatively impact students' health and well-being. During this stage of emerging adulthood, students are expected to take on greater responsibility, explore their identities, and make independent decisions all of which can be daunting and require strong adaptation skills. To support early interest and motivation, it is essential to consider students’ expectations, values, and basic needs when designing curricula and teaching methods, as these factors can greatly enhance or hinder their academic experience (Ragland et al., 2023).

At this critical time, participation in ECA can help counteract negative effects and support health, well-being, and overall quality of life. Research shows that engaging in meaningful activities contributes to the maintenance and renewal of health, making such participation essential for students’ well-being and retention (Pemberton, 2015). Numerous studies indicate that extracurricular involvement is associated with improved adaptation to university life especially through activities such as volunteering, sports, and the arts which play a vital role in managing transitional stress and are positively correlated with academic achievement (Yao et al., 2023). Furthermore, ECA plays a vital role in enhancing student experience. Research shows that, the majority of students perceive these activities as instrumental to their academic and personal success. Consequently, studies recommend that universities actively encourage and support student clubs and activities to foster a strong student community and provide opportunities for the development of social and professional skills (King et al., 2021).

It is also important to consider the challenges students face in balancing academic demands with family obligations and employment to cover tuition and living expenses. Such pressures may affect their participation in ECA. Nevertheless, research highlights positive correlations between engagement in these activities and degree completion, increased happiness and well-being, improved time management and organizational skills, as well as enhanced coping mechanisms and psychological resilience (Andrews, 2018; King et al., 2021).

With the growing number of international students in universities worldwide, findings suggest that increased participation in ECA and interaction with peers from diverse ethnic backgrounds enhance feelings of welcome and institutional belonging. Furthermore, moving from low to high involvement in ECA strengthens students’ sense of belonging. However, the impact of these interactions varies based on students’ geographical backgrounds and the language of instruction (Thies et al., 2023).

Nevertheless, ECA offers numerous benefits to educational institutions. These include increased student retention (Flores-Gonzalez, 2000), development of professional skills (Lau et al., 2014), and heightened student motivation and engagement (Gunuc and Kuzu, 2015). Munir and Zaheer (2021) found that students in open and distance learning programs experience social isolation, which can be mitigated by extracurricular educational activities that strengthen their connection to the institution, thereby reducing dropout rates.

On the other hand, ECA enhances university experience and alleviates the psychological stress of transition. Buckley and Lee (2021) note that such activities and the social networks they foster play a vital role in supporting students and reducing academic pressure. Participation in sports and physical activity has been shown to improve mental and physical health and strengthen students’ connection to the campus community, which contributes to greater university retention. Additionally, Gunuc and Kuzu (2015) reported that involvement in ECA improves students’ engagement with their institution and positively influences their sense of value and belonging. Moreover, Ndudzo (2013) highlighted that student engagement in open learning programs is influenced by communication between institutions and students, as well as social relationships among them. Thus, ECA is a key tool for fostering this communication. Institutions aiming to improve student participation, motivation, self-confidence, and employability skills will find extracurricular engagement a highly effective strategy (Munir and Zaheer, 2021).

In poultry science programs, supporting and developing ECA will have a clearly positive impact on students’ academic performance and retention, especially given the increasing diversity in their backgrounds and experiences. These activities contribute to enhancing student engagement and motivation from the outset, thereby reducing attrition rates linked to various personal, social, and economic factors. They also provide a supportive environment that complements academic learning by developing social and professional skills, fostering communication and collaboration between students and faculty members, which strengthens students’ sense of belonging and commitment. Additionally, these activities help students achieve a better balance between their academic responsibilities and personal and work commitments, improving time management, psychological resilience, and overall satisfaction with their educational experience.

### Challenges and obstacles facing the implementation of ECA in developing students' skills in poultry production and strategies for overcoming them

Studies also indicate that participation in ECAs may sometimes lead to adverse outcomes. For example, Bryant and Cantwell (2010) found that time spent by students in extracurricular clubs and organizations did not significantly enhance academic performance; instead, it showed a slight negative association with academic seriousness and higher grades. Similarly, research by Pascarella et al. (2009), and Debard et al. (2006) revealed a negative effect on cognitive development and cumulative GPA during college. Yin and Lei (2007) also reported that students who regularly participated in campus activities had lower GPAs compared to non-participants, and that increased involvement did not improve overall satisfaction with university life.

Moreover, Pinto and Ramalheira (2017) showed that graduates who engaged in ECAs tended to perform better in the labor market, regardless of academic performance. However, many students face significant barriers to participation, such as family responsibilities, time constraints, and cultural expectations (Dickinson, et al., 2021). Additional studies suggest that students lacking academic confidence may avoid participation for fear that it could detract from their studies (Harvey et al., 2017). Some students also perceive ECAs as harmful, particularly when negative experiences lead to decreased self-confidence (Dickinson et al., 2021; Griffiths et al., 2021).

Academic success, typically measured through grades and final qualifications, is a key concern for stakeholders, including students and parents. Comprehensive studies are vital for understanding how ECA participation affects academic performance. Allocating time to ECAs can detract from academic endeavors (Seow and Pan, 2014). On the other hand, Chan (2016) argued that ECAs should enhance mental well-being, support engagement, and improve academic achievement. Seow and Pan (2014) found that while ECA involvement can positively impact academic performance, excessive participation might harm it by reducing study time and focus. Hence, there is broad recognition of the need to balance ECAs with academic study and other scholarly pursuits. Additionally, ECAs offer a valuable means for developing and demonstrating competencies sought by employers (Buckley and Lee, 2021).

From an academic performance perspective, ECAs may conflict with scholarly obligations and induce psychological stress due to time demands and scheduling challenges. They may also lead to interpersonal conflicts, particularly when working with difficult individuals. Nevertheless, such challenges are often viewed as valuable learning opportunities rather than insurmountable barriers. Furthermore, ECAs contribute to the development of diverse problem-solving skills, including project management, budgeting, team leadership, and communication. Moreover, students' self-regulatory capacity, the ability to independently organize time, tasks, and priorities, has a positive influence on their overall experience (Buckley and Lee, 2021).

While academic success, defined by grades and graduation, remains a top priority for stakeholders, such as students and their families, these trends pose significant challenges for traditionally structured institutions. Rising enrollment may strain available support resources and hinder institutional adaptability, potentially leading to negative individual outcomes like isolation and loss of motivation, as well as broader institutional consequences such as lower student retention rates (Gallo, 2012).

Many studies highlight that a student's university experience and sense of belonging significantly influence post-graduation behaviors and outcomes (Iskhakova et al., 2017). ECAs are defined as "non-academic efforts organized under institutional supervision but conducted outside formal academic hours and not counted for academic credit" (Bartkus et al., 2012). These include student clubs, cultural organizations, publications, and athletic teams, providing a broad range of extracurricular opportunities. However, because ECAs are not graded or credited academically, measuring their impact remains a challenge (Seow and Pan, 2014).

To ensure high-quality education for university students, institutions must fundamentally rethink how to promote and assess student learning and development, especially by bridging the gap between academic and extracurricular experiences. In addition, HEIs should foster dynamic learning environments that encourage participation in educational experiences beyond traditional curricula and classroom settings. This holistic development approach balances academic excellence with practical workforce readiness (Ribeiro et al., 2024).

In poultry science programs specifically, it is recommended to design integrated and flexible learning pathways that combine specialized academic content with applied experiences and industry-relevant ECAs, such as field visits, cooperative training, and community-based projects. Industry stakeholders should be involved in the design of these activities to ensure alignment with market-relevant skills, thereby enhancing graduate employability, supporting ongoing curriculum improvement, and effectively linking theoretical learning to professional realities.

Furthermore, to fulfill these expectations, several recommendations should be implemented to positively impact students' academic, personal, and professional competencies, thereby improving their readiness for employment in the poultry sector. These include:

***Developing Comprehensive Training Programs***. Poultry science programs must strategically integrate ECAs into their curricula, including hands-on farm training, participation in industry-related research projects, and specialized workshops that bridge theoretical learning and practical experience. These activities make skill development engaging and contextually relevant.

***Enhancing Social and Personal Skills****.* ECAs should cultivate students' soft skills, such as teamwork, leadership, communication, and problem-solving. Participation in poultry science-related student organizations, seminars, and industry meetings supports the parallel development of academic and interpersonal skills.

***Integrating Entrepreneurship and Innovation****.* ECAs should encourage innovative thinking and entrepreneurial capability to address future challenges in poultry production. Organizing entrepreneurial workshops on sustainable agriculture and advanced technologies can help students develop market-relevant competencies.

***Strengthening Industry Partnerships***. Strong collaboration with poultry industry stakeholders is essential. Activities such as factory visits, expert-led training sessions, and professional development programs offer students real-world experience. Programs should also conduct labor market research to understand employer expectations, continuously improving academic and extracurricular offerings.

***Monitoring and Evaluating ECA Impact****.* Poultry science programs must establish mechanisms to assess the impact of ECAs on students' academic, personal, and professional development. Surveys, interviews, and performance analyses in practical activities help evaluate whether educational goals are being met.

***Tracking Student Participation and Well-being****.* Institutions should identify students at risk of burnout from excessive ECA involvement and intervene appropriately. Encouraging students to maintain a healthy balance between academic and extracurricular commitments helps ensure a well-rounded university experience.

***Embedding ECAs in Academic Schedules****.* Poultry science programs should incorporate ECAs into academic calendars and create a supportive environment that encourages student involvement in social entrepreneurship and innovation without fear of failure.

## Conclusion

In light of the growing global challenges facing the poultry industry, it has become essential to foster advanced behavioral competencies among students in poultry science programs at the higher education level extending beyond traditional technical and specialized skills. Solely focusing on technical expertise is no longer sufficient to meet the evolving demands of the labor market. This necessitates the integration of key soft skills such as collaboration, leadership, effective communication, innovation, adaptability, and problem-solving into academic curricula. Accordingly, ECA emerge as a strategic tool of soft power that contributes not only to enhancing poultry production competencies and empowering the next generation of professionals in the sector but also to improving the overall quality of poultry science programs by increasing student retention rates and attracting new enrollments. This study introduces the application of the term "soft power" as a novel concept in the context of ECA, given their indirect yet profound impact on shaping students’ character and preparing them for professional and societal engagement without imposing any form of coercion, which lies at the heart of soft power philosophy. In this context, the use of ECA as a form of soft power in poultry science programs can be defined as follows:

"Extracurricular activities are considered a form of strategic soft power and encompass a set of voluntary and complementary activities organized by colleges, academic departments, or educational programs outside the formal curriculum. These activities aim to refine communication and teamwork skills, enhance industry awareness, and develop leadership and professional competencies among poultry science students, thereby strengthening their readiness for effective and successful participation in the labor market."

Moreover, ECA serves as a strategic platform for developing behavioral and soft skills that are essential for addressing today’s pressing global challenges, including climate change, food security, and rapid urbanization. Therefore, reforming poultry science education is no longer a matter of choice, but a necessity that calls for a fundamental transformation in curriculum structure and academic practices. Additionally, integrating ECA into the educational strategy should not be regarded as an educational luxury, but rather as a foundational pillar in preparing graduates capable of contributing meaningfully to the sustainable development of the poultry industry at both local and global levels. Investing in this integrated educational approach, which combines academic learning with practical engagement through ECA, not only equips students with the competencies needed to meet the evolving demands of the poultry industry, but also enhances the competitiveness of HEIs offering poultry science programs and improves the quality of their outcomes. By cultivating students’ technical and interpersonal skills, aligning academic programs with industry needs, and promoting a culture of innovation and applied research, this approach plays a critical role in supporting the sustainable development of the poultry sector, strengthening food security, and advancing knowledge-based economic growth.

## Disclosures

The author declares that there is no conflicts of interest regarding the publication of this paper.

## Funding

This work was supported by the Deanship of Scientific Research, Vice Presidency for Graduate Studies and Scientific Research, 10.13039/501100004686King Faisal University, Saudi Arabia (Grant No. KFU252718).
